# Experimental Investigation of Mechanical and Thermal Properties of Silica Nanoparticle-Reinforced Poly(acrylamide) Nanocomposite Hydrogels

**DOI:** 10.1371/journal.pone.0136293

**Published:** 2015-08-24

**Authors:** Josergio Zaragoza, Nasim Babhadiashar, Victor O’Brien, Andrew Chang, Matthew Blanco, Aitor Zabalegui, Hohyun Lee, Prashanth Asuri

**Affiliations:** 1 Department of Bioengineering, Santa Clara University, Santa Clara, California, United States of America; 2 Department of Mechanical Engineering, Santa Clara University, Santa Clara, California, United States of America; Monash University, AUSTRALIA

## Abstract

Current studies investigating properties of nanoparticle-reinforced polymers have shown that nanocomposites often exhibit improved properties compared to neat polymers. However, over two decades of research, using both experimental studies and modeling analyses, has not fully elucidated the mechanistic underpinnings behind these enhancements. Moreover, few studies have focused on developing an understanding among two or more polymer properties affected by incorporation of nanomaterials. In our study, we investigated the elastic and thermal properties of poly(acrylamide) hydrogels containing silica nanoparticles. Both nanoparticle concentration and size affected hydrogel properties, with similar trends in enhancements observed for elastic modulus and thermal diffusivity. We also observed significantly lower swellability for hydrogel nanocomposites relative to neat hydrogels, consistent with previous work suggesting that nanoparticles can mediate pseudo crosslinking within polymer networks. Collectively, these results indicate the ability to develop next-generation composite materials with enhanced mechanical and thermal properties by increasing the average crosslinking density using nanoparticles.

## Introduction

It is well established that the addition of nanoscopic filler particles to polymer systems can have a large impact on their properties, even for very low weight fractions of nanoparticles [1–3]. Specifically, as reviewed by Tjong (2006), addition of various nanofillers including carbon nanotubes, organoclays, and inorganic nanoparticles can significantly improve the mechanical performances of both amorphous and semicrystalline polymer composites [2]. Improvements in polymer properties are not limited to mechanical characteristics but also extend to thermal and electrical properties[4–9] as well as chemical resistance [10, 11]. Furthermore, similar enhancements in material properties have also been reported for elastomers and hydrogels as well [12–18]. Literature that reviews the properties of hydrogel nanocomposites are especially relevant to the scope this paper as defined by the choice of our model polymer system.

Several experimental and theoretical studies support that the enhancements in polymer properties are dependent on the polymer and nanofiller surface chemistry as well as the dispersion quality of the nanofillers [19–23]. Research efforts are currently focused on structure–property relationships of polymer nanocomposites, as well as in quantifying the role of nanofiller/matrix interfacial interactions on nanocomposite properties, to further shed light on the mechanisms behind these reinforcements [24–28]. One hypothesis that has gained significant support in recent years is that strong polymer/nanofiller interactions facilitate the formation of non-covalent or pseudo crosslinks, and thereby contribute to enhancements in polymer properties [18, 29–33]. In this study, our initial objective was to conduct experiments in further support of the pseudo crosslinking hypothesis using synthetic polyacrylamide (pAAM) hydrogels incorporating silica nanoparticles (SiNPs) as the model system. Both these materials have been well characterized, are commercially available, and are routinely used in a wide variety of industrial and scientific applications. Moreover, the degree of crosslinking has a significant effect on hydrogel swelling properties, which allows us to interrogate the pseudo crosslinking hypothesis. Mechanical properties of the hydrogel nanocomposites were characterized through measurements of viscoelastic and compressive moduli. We also explored if pAAm-SiNP hydrogels exhibited improved thermal properties, investigated using the laser flash technique, relative to neat polyacrylamide hydrogels, as well as if there were any correlations between the enhancements in pAAm mechanical and thermal properties afforded due to the addition of silica nanoparticles. Since the speed of heat propagation in a solid is dependent on its elastic modulus, it can be expected that any changes in elastic moduli due to the addition of nanoparticles will lead to enhancement in the thermal transport properties of the hydrogels beyond the values anticipated by traditional effective medium theories.

Our data, obtained using silica nanoparticle–pAAm hydrogel composites as the model system, showed significant enhancements in the elastic and compressive properties of hydrogels upon the addition of nanoparticles, consistent with previous investigations of polymer nanocomposite mechanical properties. Furthermore, our experiments revealed decreased swellability of pAAm nanocomposites relative to neat pAAm hydrogels, thereby demonstrating a strong role of pseudo crosslinking mediated by polymer-nanoparticle interactions on the observed enhancements in mechanical properties. Moreover, our results demonstrated a high correlatability between the enhancements in mechanical and thermal properties of the pAAm-SiNP composites, thereby suggesting that SiNP-mediated pseudo crosslinking of pAAm hydrogel can also lead to enhancements in its thermal properties. These results therefore indicate that the approach of using nanofillers to improve mechanical properties may also be used to engineer thermal properties of hydrogels.

## Materials and Methods

### Materials

The monomer and crosslinker solutions, acrylamide (AAm, 40% w/v) and N,N′-methylenebis(acrylamide) (Bis, 2% w/v), as well as ammonium persulfate (APS, initiator) and N,N,N′,N′-tetramethylethylenediamine (TEMED, catalyst) were purchased from Sigma Aldrich (St. Louis, MO) and used as received. Tris-HCl buffer was obtained from Life Technologies (Carlsbad, CA). Bindzil silica nanoparticle colloid solutions with mean particle sizes of 4, 20, and 100 nm, were obtained as a gift from AkzoNobel Pulp and Performance Chemicals Inc. (Marietta, GA). (See S1 Table for physicochemical properties of the silica nanoparticles as provided by the supplier.)

### Polymerization reaction

To prepare chemically crosslinked polyacrylamide hydrogels, AAm and Bis stocks were diluted to their desired concentrations in pH 7.2, 250 mM Tris-HCl buffer, followed by the addition of 10 μL of freshly prepared APS solution and 1 μL of TEMED to initiate the reaction. Final AAm and Bis concentrations were 10% w/v and 0.5% w/v respectively, and final reaction volume was 1 mL. For the nanoparticle experiments, various amounts of silica nanoparticles were added the reaction mixture before the addition of APS and TEMED. The polymerization reactions were performed at 25°C, with minimal exposure to air as oxygen inhibits the reaction.

### Rheological measurements

Rheological measurements of the pAAm gels with and without nanoparticles were carried out using a MCR302 rotational rheometer (Anton Paar, Austria) using parallel plate geometry. Immediately following the addition of APS and TEMED, 200 μL of the well-mixed reaction mixture was pipetted onto the lower plate. The upper plate was lowered to the desired sample thickness of 1 mm. Amplitude sweeps at a constant frequency of 1 Hz were carried then out to ensure that the measurements were performed in the linear viscoelastic regimes for the hydrogel samples. Next, dynamic sweep tests over frequencies ranging from 0.1–100 Hz were recorded in the linear viscoelastic regimes (strain amplitude = 0.01) to determine the shear storage modulus, G’. The shear storage modulus relates to the elastic behavior of a viscoelastic material and is a measure of its stiffness.

### Compressive modulus measurements

For the compressive modulus measurements, we first prepared pAAm hydrogel disks using an acrylic mold (1.6 mm thick and 6.5 mm in radius); see S1 Fig for a detailed description. Briefly, immediately following the addition of APS and TEMED, 210 μL of the well-mixed reaction mixture, either containing or not containing nanoparticles, was pipetted into individual molds. After waiting for 1 hour to ensure complete gelation (gelation usually occurs within 20 minutes), the hydrogel discs were taken from the mold and gently wiped with tissue paper to remove any excess water before performing the measurements. The compressive modulus measurements were tested at room temperature under unconfined conditions using the Mach-1 mechanical testing system (Biomomentum, Canada). The pAAm gel disks prepared using the acrylic molds were compressed at 0.1 mm/s to 50% of sample thickness, and the compressive modulus was determined by calculating the slope of the linear region of the stress-strain curves (typically between 10–15% strain).

### Measurement of the swelling properties

To study the swelling properties of the pAAm nanocomposites, the hydrogel disks prepared as described above were wiped with tissue paper to remove any excess water, weighed and then immersed in pH 7.2, 100 mM Tris-HCl buffer. Hydrogel samples were withdrawn from the buffer at different time intervals and their weights were determined after first blotting excess buffer with tissue paper; the swelling experiments were carried out for 24 hours. The swelling ratios at different time intervals were then calculated using the following equation:

$$Swelling\;ratio\;\left(\%\right)=\;\left[\frac{W_{t}-W_{0}}{W_{0}}\right]\times 100$$ (1)

where *W*_*t*_ is the weight of pAAm gel samples at a given time interval and *W*_*0*_ is the initial weight (before immersing in buffer).

### Thermal diffusivity measurements

Thermal diffusivities of the hydrogel samples were performed using the commercially available Netzsch LFA 457 Laser Flash System (Burlington, MA), based on a corrected Cape-Lehman method [34]. Thermal diffusivity measurements can be carried out within ±3%. The pAAm hydrogel disk samples were prepared with or without nanoparticles, as described above, and any excess water was removed before the measurement. The surface was coated with black graphite spray to improve signal to noise ratio.

## Results

### Nanoparticle mediated enhancement in hydrogel mechanical properties

**Fig 1:**
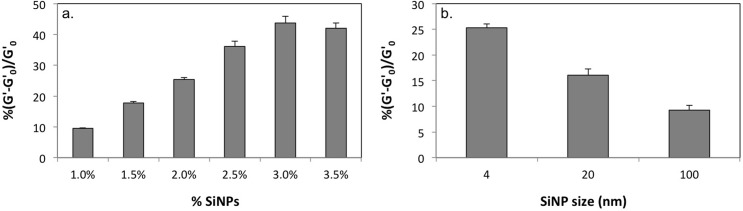
Viscoelastic properties of pAAm hydrogel nanocomposites. Percent relative elastic moduli of pAAm hydrogels as a function of silica nanoparticle (a) concentration (prepared using 4 nm SiNPs) and (b) size (prepared using a final concentration of 2% w/v SiNPs). The values for relative elastic modulus were calculated by normalizing the values for pAAm-SiNP nanocomposite gels (G’) to those for neat pAAm gels (G’_0_). Error bars indicate the standard deviation of triplicate measurements.

**Fig 2:**
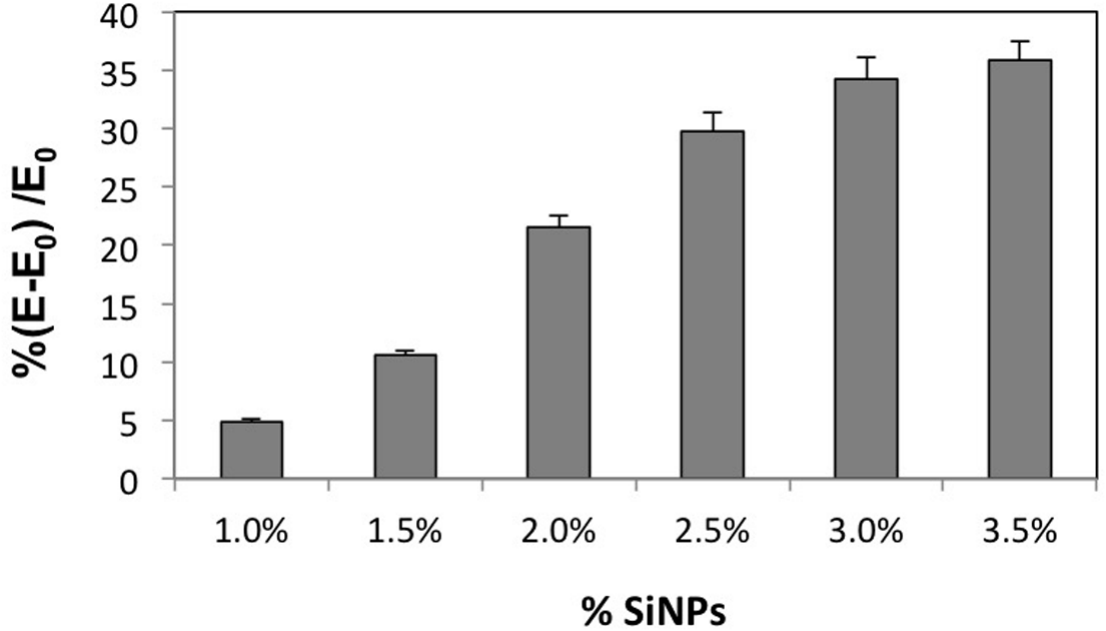
Compressive modulus of pAAM hydrogel nanocomposites. Percent relative compressive moduli of pAAm hydrogels as a function of 4 nm silica nanoparticle concentration. The values for relative compressive modulus were calculated by normalizing the values for pAAm-SiNP nanocomposite gels (E) to those for neat pAAm gels (E_0_). Error bars indicate the standard deviation of triplicate measurements.

We used poly(acrylamide) (pAAm) hydrogel, a linear chain polymer of repeating acrylamide units crosslinked using N,N'-methylenebisacrylamide (Bis), incorporating commercially available amorphous silica nanoparticles (SiNPs) of various sizes, as the model system. Experimental investigations of the nanocomposite mechanical properties using rotational rheometry revealed that the elastic modulus (G’) of pAAm-SiNP composites relative to the neat polymer was strongly dependent on the nanoparticle concentration (Fig 1A and S2 Fig). Additionally, we observed an upper limit to the gains in the elastic modulus due to addition of nanoparticles (Fig 1A). We also explored the effects of nanoparticle size on the elastic modulus of pAAm-SiNP composites using 4, 20, and 100 nm-sized SiNPs. Interestingly, these studies indicated that the enhancements in pAAm elastic modulus afforded by the addition of SiNPs decreased with increasing nanoparticle size (Fig 1B and S2 Fig). Similar results, i.e. decrease in polymer nanocomposite mechanical properties for larger nanoparticles, have been reported in previous literature [35]. Additionally, we determined the equilibrium unconfined compressive moduli of the pAAm-SiNP composites incorporating SiNPs of various concentrations and sizes to complement the rheological analyses. The compressive moduli experiments also clearly demonstrated strong dependence of the nanocomposite mechanical properties on nanoparticle size and concentration, with similar trends as those observed for rotational rheometry experiments (Fig 2 and S3 Fig).

### Increase in average crosslinking density mediated by polymer-nanoparticle interactions

**Fig 3:**
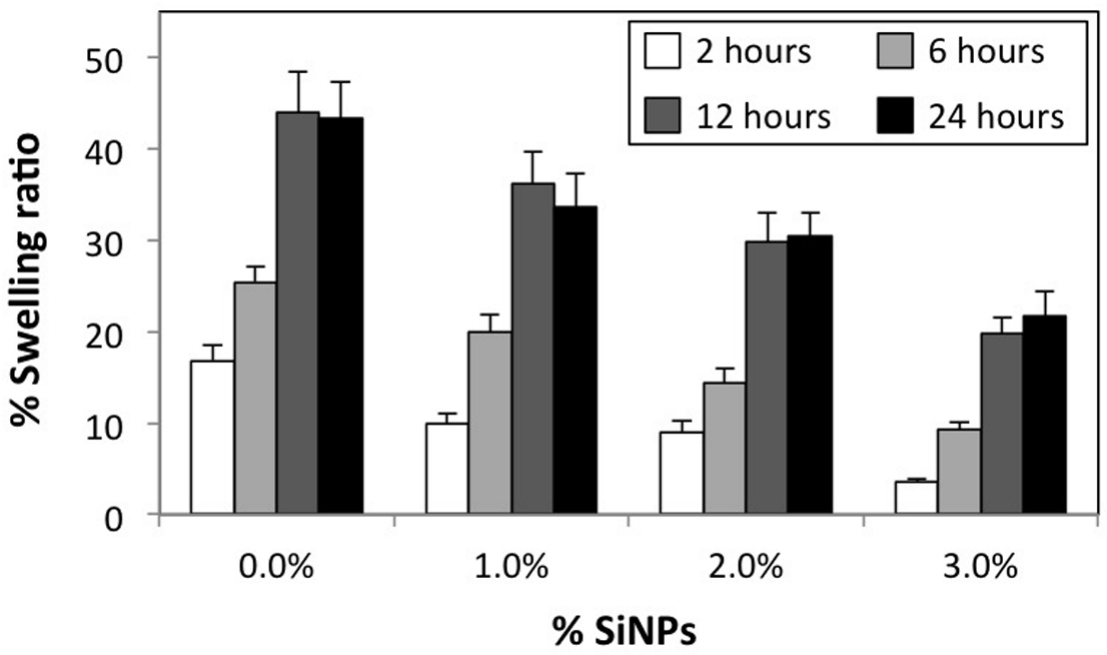
Swelling properties of pAAM hydrogel nanocomposites. Percent swelling ratios of pAAm hydrogels as a function of 4 nm silica nanoparticle concentration at various time points – 2 hours (white bars), 6 hours (light grey bars), 12 hours (dark grey bars), and 24 hours (black bars). The values for swelling ratio were calculated by normalizing the values obtained for pAAm gels either containing or not containing SiNPs at various time points to those obtained at time = 0 min. Error bars indicate the standard deviation of triplicate measurements.

As described earlier, previous studies have suggested that nanoparticles can act as pseudo crosslinkers and contribute to the extent of crosslinking in the polymer network, and thereby facilitate reinforcements in mechanical properties. Also, it has already been demonstrated that hydrogen bonding promotes strong interactions between pAAm chains and SiNP surface [36, 37]. We therefore wished to test the hypothesis that the observed increases in elastic and compressive moduli of pAAm nanocomposites relative to the neat polymer is afforded by increased average crosslinking density mediated by pAAm-SiNP interactions. We compared the swellability of pAAm-SiNP gels to neat pAAm gels; if pAAm-SiNP interactions contribute to an increase in average polymer crosslinking density, the swellability of pAAm-SiNP gels should be lower than that of neat pAAm gels. Fig 3 compares the swellability of neat pAAm gels and pAAm gels incorporating various concentrations of 4 nm SiNPs. The data indicated that the swelling for both neat pAAm and pAAm incorporating SiNPs saturates after ca. 12 hours. Moreover, decreased swellability of pAAm nanocomposites with increasing SiNP concentration strongly suggests the role of pAAm-SiNP interactions in facilitating an increase in the average crosslinking density of the hydrogel network, and thereby decreased swellability of pAAm-SiNP gels relative to neat gels, as well as SiNP mediated enhancement in pAAM hydrogel mechanical properties.

### Enhanced thermal diffusivities of the pAAm hydrogel nanocomposites

Next, we proceeded to assess the effect of the addition of nanoparticles on thermal properties of pAAm hydrogels. Although hydrogels are not traditionally used for the analysis of thermal properties of polymer nanocomposites, we were interested in the use of our model system as a test of the kinetic theory:[38] thermal conductivity (*k*) of a solid is proportional to its sound velocity (*v*), which is in turn proportional to the square root of the elastic modulus (*E*):

$$k=\;\rho C\alpha =\;\frac{1}{3}Cv\Lambda$$ (2)

$$v=\;\sqrt{\frac{E}{\rho }}$$ (3)

where ρ is the density, C is heat capacity, α is thermal diffusivity, and Λ is the phonon mean free path of phonon. Eqs (2) and (3) can be combined to describe the dependence of thermal diffusivity on elastic moduli:

$$\alpha \propto \sqrt{E}$$ (4)

Changes in the thermal diffusivity of the polymer due to the addition of nanoparticles can be caused by nanoparticle-mediated changes in any of the three properties (i.e. changes in either *ρ*, *E*, or Λ). However, since we are adding a small amount of nanoparticles, changes in density (*ρ*) can be relatively ignored. Further, if we assume that the phonon mean free path (Λ) is not affected by the low volume fraction of nanoparticles, change in the thermal diffusivity of a polymer pseudo-crosslinked using nanoparticles should be directly proportional to the square root of the change in its elastic moduli (*E*). This assumption is reasonable for our system, as the phonon mean free path of polymer chain is usually small, and the diffuse scattering due to impurities should not significantly affect the mean free path, as described by Matthiessen's rule [39].

**Fig 4:**
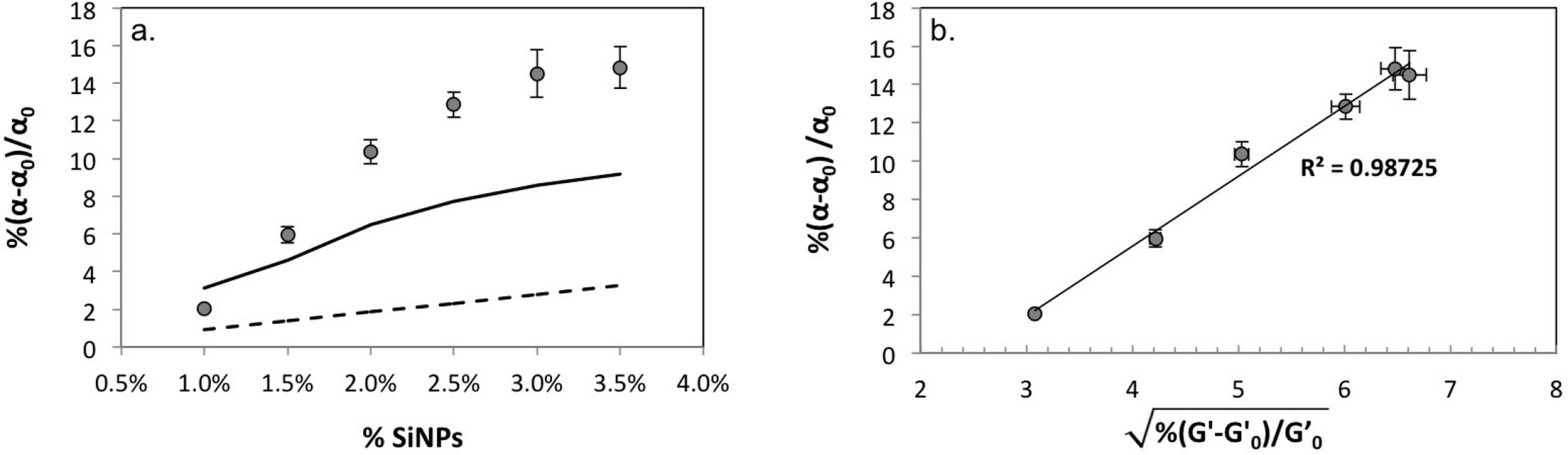
Thermal properties of pAAM hydrogel nanocomposites. (a) Percent relative thermal diffusivities of pAAm hydrogels as a function of 4 nm silica nanoparticle concentration (grey circles), compared to the traditional effective medium theory (solid line), and the modified effective medium theory (dashed line). The values for relative thermal diffusivity were calculated by normalizing the values for pAAm-SiNP nanocomposite gels (α) to those for neat pAAm gels (α_0_). Error bars indicate the standard deviation of triplicate measurements. (b) Correlation between percent relative enhancement in thermal diffusivities and the square root of percent relative enhancement in the elastic moduli for pAAm-SiNP composites (prepared using 4 nm SiNPs). Relative enhancements in thermal diffusivity and elastic modulus were calculated as previously described. Error bars indicate the standard deviation of triplicate measurements.

Fig 4A shows the observed relative enhancements in thermal diffusivity of pAAm hydrogel nanocomposites as a function of the weight fraction of the silica nanoparticles. The data from Fig 4A and S3 Fig clearly shows relative enhancements as a function of nanoparticle size; the trends observed for enhancements in the thermal diffusivity are similar to those observed for the mechanical properties of pAAm-SiNP composites, with a high degree of correlation between the enhancement in thermal diffusivity and the square root of the enhancement in the elastic moduli (Fig 4B). Moreover, both mechanical and thermal reinforcements exhibited similar saturation behaviors, with plateauing occurring near 3% SiNPs.

One should note that this is not the first attempt to relate enhancement of thermal transport properties of polymer nanocomposites with the changes in elastic moduli. However, majority research works so far have attempted to explain enhanced thermal conductivity using the simple effective medium theory (EMT) without full consideration of the change in the thermal conductivity of a base medium [40]. Based on the most widely adopted EMT [41], the thermal conductivity of composite materials can be written as:

$$k_{eff}=\frac{k_{p}+2k_{b}+2\left(k_{p}-k_{b}\right)\varphi }{k_{p}+2k_{b}-\left(k_{p}-k_{b}\right)\varphi }k_{b}$$ (5)

where *k*_*b*_ is the thermal conductivity of the base material, *k*_*p*_ is the thermal conductivity of the added nanoparticles, and φ is the nanoparticle volume fraction. Using Eq (2), Eq (5) can be written as,

$${\left(\rho C\alpha \right)}_{eff}=\frac{{\left(\rho C\alpha \right)}_{p}+2{\left(\rho C\alpha \right)}_{b}+2\left[{\left(\rho C\alpha \right)}_{p}-{\left(\rho C\alpha \right)}_{b}\right]\varphi }{{\left(\rho C\alpha \right)}_{p}+2{\left(\rho C\alpha \right)}_{b}-\left[{\left(\rho C\alpha \right)}_{p}-{\left(\rho C\alpha \right)}_{b}\right]\varphi }{\left(\rho C\alpha \right)}_{b}$$ (6)

Since the product of density and heat capacity is in the same order of magnitude for most materials, and the value does not change significantly for low concentration of nanoparticles, Eq (6) can also be written as:

$$\alpha_{eff}=\frac{\alpha_{p}\frac{{\left(\rho C\right)}_{p}}{{\left(\rho C\right)}_{b}}+2\alpha_{b}+2\left(\alpha_{p}\frac{{\left(\rho C\right)}_{p}}{{\left(\rho C\right)}_{b}}-\alpha_{b}\right)\varphi }{\alpha_{p}\frac{{\left(\rho C\right)}_{p}}{{\left(\rho C\right)}_{b}}+2\alpha_{b}-\left(\alpha_{p}\frac{{\left(\rho C\right)}_{p}}{{\left(\rho C\right)}_{b}}-\alpha_{b}\right)\varphi }\alpha_{b}$$ (7)

From Fig 4A, it is clear that the observed values of enhancement in thermal diffusivity due the addition of nanoparticles are much larger than those calculated using the traditional EMT (black dashed line in Fig 4A). The traditional EMT assumes that the base thermal diffusivity does not change due to the addition of particles. Thermal diffusivity of the base material is expected to change due to crosslinking, and therefore should be modified before applying the EMT. The *α*_*b*_ terms in Eq 7 can be multiplied by the square root of enhancement ratio (*r*) of elastic moduli to obtain the equation for the modified EMT:

$$\alpha_{eff}=\frac{\alpha_{p}\frac{{\left(\rho C\right)}_{p}}{{\left(\rho C\right)}_{b}}+2\sqrt{r}\alpha_{b}+2\left(\alpha_{p}\frac{{\left(\rho C\right)}_{p}}{{\left(\rho C\right)}_{b}}-\sqrt{r}\alpha_{b}\right)\varphi }{\alpha_{p}\frac{{\left(\rho C\right)}_{p}}{{\left(\rho C\right)}_{b}}+2\sqrt{r}\alpha_{b}-\left(\alpha_{p}\frac{{\left(\rho C\right)}_{p}}{{\left(\rho C\right)}_{b}}-\sqrt{r}\alpha_{b}\right)\varphi }\sqrt{r}\alpha_{b}$$ (8)

Fig 4A also shows the modified EMT (black solid line). Although the modified EMT shows more enhancement than the traditional EMT, it cannot still fully explain the anomalous enhancement in the experimental results. The differences in enhancements between the experimental results and the modified EMT may be attributed to the secondary enhancement in heat capacity or mean free path due to modified phonon density of states. Further studies on phonon dispersion relation and change in mean free path may be needed to fully understand its enhancement.

## Conclusions

In this work, we performed experiments to further our understanding of the effects of nanoparticles on hydrogel material properties. The model system, pAAM hydrogels incorporating silica nanoparticles, was evaluated by measurements of three different polymer properties: elastic modulus, swellability, and thermal diffusivity. Results from the experimental analyses showed that both hydrogel mechanical and thermal properties are significantly dependent on the size and concentration of silica nanoparticles; there also seems to be an upper limit to the gains in polymer properties due to addition of nanoparticles. Moreover, we observed that the swellability of pAAm-SiNP hydrogels was significantly lower than that of neat pAAm gels. Our data also presented a strong correlation between enhancements in viscoelastic moduli and thermal diffusivity, thus indicating that nanofiller-mediated enhancements in polymer structure can also translate into enhancements in its thermal properties. The outcomes of these results are two-fold. First, our data demonstrate that the addition of nanoparticles can lead to higher hydrogel crosslinking densities, thereby lending strong support to the hypothesis that pseudo crosslinking can significantly contribute to enhancements in mechanical properties of polymer nanocomposites. Second, our experiments not only demonstrated correlatability between enhancements in mechanical and thermal properties of hydrogel nanocomposites, but also anomalous enhancements in thermal diffusivity upon the addition of nanoparticles, beyond the values predicted by the effective medium theory, thus indicating that nanofiller-mediated enhancements in polymer structure can also translate into enhancements in its thermal properties. Collectively, these results suggest a new direction to engineer thermal properties of polymers.

## Supporting Information

S1 Fig Preparation of pAAm hydrogel disks. (a) Dimensions of the acrylic mold used for the preparation of pAAm hydrogel disks. (b) In Step 1, 210 μL of the reaction mixture, either containing or not containing nanoparticles, was pipetted into individual molds. A glass slide was placed on the samples to limit exposure to oxygen, which inhibits the polymerization reaction (Step 2). After waiting for 1 hour to ensure complete gelation, the hydrogel discs were taken from the mold for further testing (Step 3).
(PDF)

S2 Fig Viscoelastic properties of pAAM hydrogel nanocomposites. Representative plots showing the dependence of the viscoelastic properties of pAAM hydrogel nanocomposites on silica nanoparticle (a) concentration (prepared using 4 nm nanoparticles) – 1% w/v (blue line), 2% w/v (green line), and 3% w/v (red line) and (b) size (using a final concentration of 2% w/v nanoparticles) – 100 nm (blue line), 20 nm (green line), and 4 nm (red line). Control hydrogels without nanoparticles is shown in grey.
(PDF)

S3 Fig Mechanical and thermal properties of pAAM hydrogel nanocomposites. Percent relative enhancements in elastic modulus (white bars), compressive modulus (grey bars), and thermal diffusivity (black bars) of pAAm hydrogel nanocomposites as a function of silica nanoparticle size. Relative enhancements in the various mechanical and thermal properties were calculated as described in the Methods section. Error bars indicate the standard deviation of triplicate measurements.
(PDF)

S1 Table Properties of the silica nanoparticles as provided by the supplier AkzoNobel Pulp and Performance Chemicals Inc. (Marietta, GA). (PDF)
