# Supplementary material for: Experimental Investigation of Mechanical and Thermal Properties of Silica Nanoparticle-Reinforced Poly(acrylamide) Nanocomposite Hydrogels
Source: PLoS One. 2015 Aug 24;10(8):e0136293. doi: 10.1371/journal.pone.0136293 (PMC4547727; doi:10.1371/journal.pone.0136293)

Experimental investigation of mechanical and thermal properties of silica nanoparticle-reinforced poly(acrylamide) nanocomposite hydrogels

*Corresponding authors: hlee@scu.edu and asurip@scu.edu

S1 Fig. Preparation of pAAm hydrogel disks.

(a) Dimensions of the acrylic mold used for the preparation of pAAm hydrogel disks. (b) In Step 1, 210 µL of the reaction mixture, either containing or not containing nanoparticles, was pipetted into individual molds. A glass slide was placed on the samples to limit exposure to oxygen, which inhibits the polymerization reaction (Step 2). After waiting for 1 hour to ensure complete gelation, the hydrogel discs were taken from the mold for further testing (Step 3).

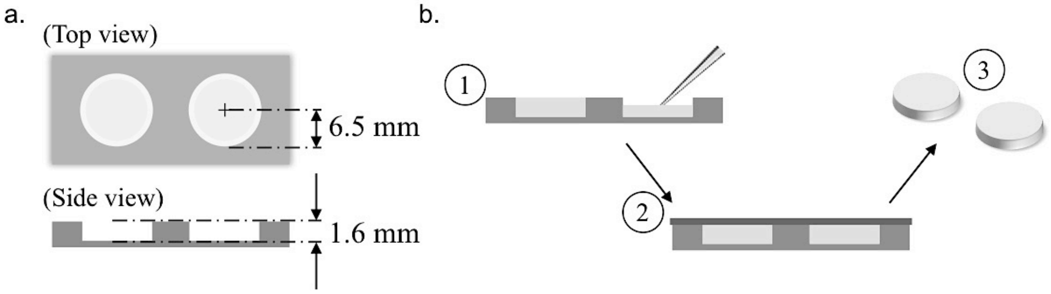

Supplement: S1 Fig — (a) Dimensions of the acrylic mold used for the preparation of pAAm hydrogel disks. (b) In Step 1, 210 μL of the reaction mixture, either containing or not containing nanoparticles, was pipetted into individual molds. A glass slide was placed on the samples to limit exposure to oxygen, which inhibits the polymerization reaction (Step 2). After waiting for 1 hour to ensure complete gelation, the hydrogel discs were taken from the mold for further testing (Step 3). (PDF) [file pone.0136293.s001.pdf]
