# Supplementary material for: Experimental Investigation of Mechanical and Thermal Properties of Silica Nanoparticle-Reinforced Poly(acrylamide) Nanocomposite Hydrogels
Source: PLoS One. 2015 Aug 24;10(8):e0136293. doi: 10.1371/journal.pone.0136293 (PMC4547727; doi:10.1371/journal.pone.0136293)

Experimental investigation of mechanical and thermal properties of silica nanoparticle-reinforced poly(acrylamide) nanocomposite hydrogels

*Corresponding authors: hlee@scu.edu and asurip@scu.edu

S2 Fig. Viscoelastic properties of pAAM hydrogel nanocomposites.

Representative plots showing the dependence of the viscoelastic properties of pAAM hydrogel nanocomposites on silica nanoparticle (a) concentration (prepared using 4 nm nanoparticles) – 1% w/v (blue line), 2% w/v (green line), and 3% w/v (red line) and (b) size (using a final concentration of 2% w/v nanoparticles) – 100 nm (blue line), 20 nm (green line), and 4 nm (red line). Control hydrogels without nanoparticles is shown in grey.

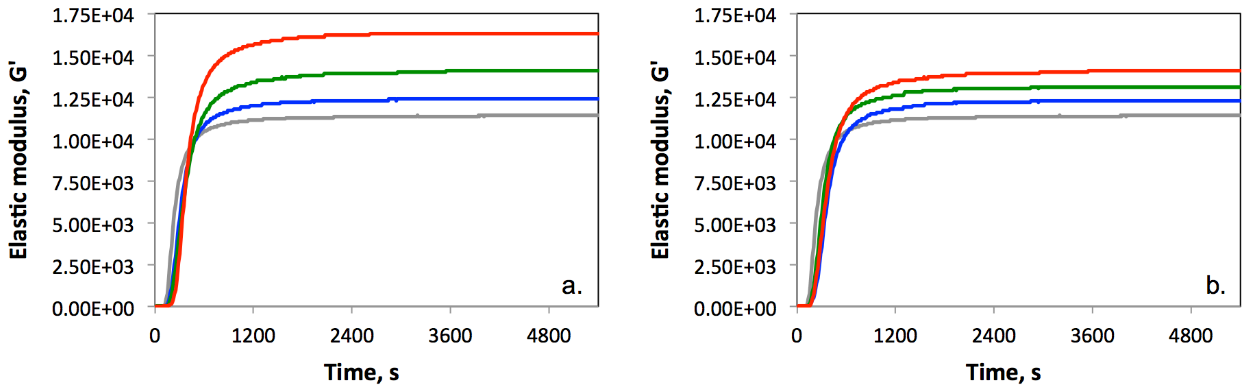

Supplement: S2 Fig — Representative plots showing the dependence of the viscoelastic properties of pAAM hydrogel nanocomposites on silica nanoparticle (a) concentration (prepared using 4 nm nanoparticles) – 1% w/v (blue line), 2% w/v (green line), and 3% w/v (red line) and (b) size (using a final concentration of 2% w/v nanoparticles) – 100 nm (blue line), 20 nm (green line), and 4 nm (red line). Control hydrogels without nanoparticles is shown in grey. (PDF) [file pone.0136293.s002.pdf]
