# Supplementary material for: Experimental Investigation of Mechanical and Thermal Properties of Silica Nanoparticle-Reinforced Poly(acrylamide) Nanocomposite Hydrogels
Source: PLoS One. 2015 Aug 24;10(8):e0136293. doi: 10.1371/journal.pone.0136293 (PMC4547727; doi:10.1371/journal.pone.0136293)

Experimental investigation of mechanical and thermal properties of silica nanoparticle-reinforced poly(acrylamide) nanocomposite hydrogels

*Corresponding authors: hlee@scu.edu and asurip@scu.edu

S3 Fig. Mechanical and thermal properties of pAAM hydrogel nanocomposites.

Percent relative enhancements in elastic modulus (white bars), compressive modulus (grey bars), and thermal diffusivity (black bars) of pAAm hydrogel nanocomposites as a function of silica nanoparticle size. Relative enhancements in the various mechanical and thermal properties were calculated as described in the Methods section. Error bars indicate the standard deviation of triplicate measurements.

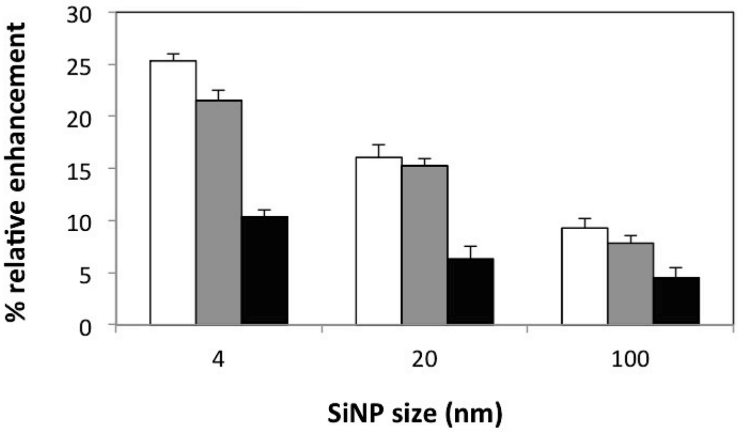

Supplement: S3 Fig — Percent relative enhancements in elastic modulus (white bars), compressive modulus (grey bars), and thermal diffusivity (black bars) of pAAm hydrogel nanocomposites as a function of silica nanoparticle size. Relative enhancements in the various mechanical and thermal properties were calculated as described in the Methods section. Error bars indicate the standard deviation of triplicate measurements. (PDF) [file pone.0136293.s003.pdf]
