# Supplementary material for: Experimental Investigation of Mechanical and Thermal Properties of Silica Nanoparticle-Reinforced Poly(acrylamide) Nanocomposite Hydrogels
Source: PLoS One. 2015 Aug 24;10(8):e0136293. doi: 10.1371/journal.pone.0136293 (PMC4547727; doi:10.1371/journal.pone.0136293)
Supplement: S1 Table — (PDF) [file pone.0136293.s004.pdf]

Experimental investigation of mechanical and thermal properties of silica nanoparticle-reinforced poly(acrylamide) nanocomposite hydrogels

*Corresponding authors: hlee@scu.edu and asurip@scu.edu

S1 Table.

Properties of the silica nanoparticles as provided by the supplier AkzoNobel Pulp and Performance Chemicals Inc. (Marietta, GA).

Sample	Particle size (mean, nm)	Particle concentration (% wt/vol)	Particle concentration (number/mL)
Bindzil 215	4	15	2.3×10^{18}
Bindzil 2040	20	40	5.9×10^{16}
Bindzil 9950	100	50	6.4×10^{14}
